# Visual self-motion information contributes to passable width perception during a bike riding situation

**DOI:** 10.3389/fnins.2022.938446

**Published:** 2022-07-22

**Authors:** Naoki Kuroda, Kazuhiro Ikeda, Wataru Teramoto

**Affiliations:** ^1^Graduate School of Social and Cultural Sciences, Kumamoto University, Kumamoto, Japan; ^2^Japan Society for the Promotion of Science, Tokyo, Japan; ^3^Faculty of Letters, Kumamoto University, Kumamoto, Japan

**Keywords:** obstacle avoidance, perceived passable width, self-motion, spatial perception, virtual reality

## Abstract

Previous studies have shown that space perception around the body is altered by self-motion, and that several self-motion cues from different modalities, including vision, proprioception, the vestibular system, and the motor system (motor commands) contribute to it. However, studies on how online self-motion information affects the perception of a passable width of a narrow aperture is largely overlooked by existing literature. Therefore, this study investigated this issue during virtual bike riding. Participants observed a narrow doorway aperture with varied widths in a virtual environment through a head-mounted display while riding a stationary bike. Visual self-motion information was presented by optical flow, while motor commands and proprioceptive feedback (non-visual information) was provided by having participants pedal the bike. The participants were then required to judge whether the presented aperture was passable. Experiment 1, where both visual and non-visual cues were provided, confirmed that the perceived passable width significantly increased with increasing self-motion speed, as previously shown during walking. Experiment 2, where self-motion cues were manipulated, showed that expansion of the perceived passable width was mainly induced by visual self-motion information. These results suggest that online self-motion information can affect passable width perception during bike riding and that visual self-motion information plays a significant role in this perception.

## Introduction

When one interacts with the external environment, there is a need to appropriately perceive whether the action to be attempted aligns with the environmental constraints. For example, if an object in front of the observer is perceived to be unreachable, he/she would walk to the object, reach with some tool, or ask someone to bring it to them. If the width of a passageway aperture is perceived to be narrower than the observer’s shoulder width to pass through, he/she would approach the aperture by turning their shoulder or give up on passing through the aperture. Thus, the perception of action possibility constrained by the surrounding environment (i.e., affordance perception, Gibson, 1979) plays an important role in determining upcoming action.

Perception of passable width of aperture (i.e., narrow space formed between obstacles such as walls, cars, and other people) has been an intensively investigated research question. In a seminal study by Warren and Whang (1987), participants were tasked to walk through apertures with various widths, and the minimum width at which participants turned their shoulders instead of walking straight was measured (Experiment 1). They found that the minimum width was constant, which was 1.3 times more than the participants’ shoulder width when they walked naturally. Values greater than 1 mean that a certain safety margin is allowed for body sway and error. They also showed that this ratio increased with walking speed. Furthermore, in their Experiment 2, the perceived passable width was significantly smaller when it was estimated from a distance than that during actual walking (Experiment 1). Although the authors argue that the differences between Experiments 1 and 2 were due to the wording of instructions (“walk naturally” in Experiment 1 and “judge whether they could walk straight through the opening without turning their shoulder” in Experiment 2), these results raise a possibility that the perceived passable width can be changed depending on online self-motion information.

Forms of locomotion on land is not limited to walking or running. It can be realized through vehicles. Thus far, passable width perception in vehicles has been investigated in wheelchair users (Savelsbergh et al., 1998; Higuchi et al., 2004, 2009) and fire engine drivers (Kroll and Crundall, 2019). Higuchi and his colleagues demonstrated that novice wheelchair users underestimated the critical aperture width to pass through even after the 8-day training (Higuchi et al., 2004). Conversely, expert wheelchair users accurately estimated the spatial requirements for wheelchair use even when using an unfamiliar wheelchair (i.e., a wheelchair which was not owned by the individual) (Higuchi et al., 2009). Kroll and Crundall (2019) targeted fire engine drivers, whose cars were particularly large. They showed that experienced fire engine drivers accurately estimated whether to proceed or not when narrow apertures were presented, whereas novice fire engine drivers did not. It is therefore probable that passable width perception is accurate when participants are skilled with the given vehicle locomotion. However, it is worth noting that no self-motion information was presented in these studies when participants estimated the passability of the aperture. In the study by Higuchi et al. (2004), participants were allowed to use the wheelchair before a visual estimation task. However, during the visual estimation task, they viewed the aperture from a distance and judged whether they could pass through the aperture while sitting in the wheelchair. In the study by Kroll and Crundall (2019), participants viewed a computer monitor which presented video clips recorded beforehand from the drivers’ viewpoint, to judge whether they could pass through the aperture created by two vehicles and/or road furniture. Thus, since no online self-motion information was presented during these tasks, the extent as to which the participants could accurately imagine the situation based on their past experiences might have had a critical influence on passable width perception. Considering the differences between Experiments 1 and 2 in Warren and Whang’s (1987) study, the absence of online self-motion information could possibly affect performance.

Given all this, this study aimed to investigate the effects of online self-motion information on the perception of passable width in vehicle locomotion using a virtual reality (VR) bike riding situation. There are two reasons why bike riding was focused. First, a bike is a very popular commuting vehicle in the region of Kumamoto, Japan. As a result, we can investigate the performance of average (either novice or very skilled) individuals who are familiar with this locomotion tool. Second, the VR system can operationally reproduce visual and non-visual self-motion information during bike riding, except for vestibular information in acceleration/deceleration. For our study, Experiment 1 was designed to investigate whether self-motion speed affected the passable width perception. Self-motion speed variable is one of the variables affecting passable width perception during walking (Warren and Whang, 1987). We conducted this experiment to confirm the generalizability of the previous finding to a bike riding situation. The results showed that the passable width increased with increase in self-motion speed, as previously shown during walking (Warren and Whang, 1987). Experiment 2 investigated how visual and non-visual (motor command and proprioceptive feedback) self-motion cues, which were the two main self-motion cues during VR bike riding, contributed to passable width perception. It should be noted that non-visual self-motion cues in this study only included motor commands and proprioceptive feedback. Previous studies showed that visual self-motion information dominated over non-visual information during bike riding in traveled distance perception (Sun et al., 2004), whereas this relationship was reversed during walking (Harris et al., 2000; Campos et al., 2012, 2014). This difference is related to the fact that, although self-motion speed and its direction are closely associated with non-visual information related to the lower limbs during walking, they are not always associated in a bike riding situation. This is because non-visual information of the lower limbs can change depending on bike gear, and the movement direction of the bike is linked to the direction of the front wheel. The results of Experiment 2 were consistent with the previous study on traveled distance perception during bike riding.

## Experiment 1

### Methods

#### Participants

A total of 24 undergraduate students were included [10 females; mean age: 21.6 ± 0.8 (*SD*) years]. All participants reported normal or corrected-to-normal vision, normal touch sensation, and no vestibular-system diseases. All were naïve on the purpose of the experiment. This study was approved by the Ethics Committee of the Graduate School of Humanities and Social Sciences, Kumamoto University and was conducted in accordance with the principles of the Declaration of Helsinki (1964). Written informed consent was obtained from all participants before they commenced the experiment.

#### Apparatus and stimuli

**FIGURE 1:**
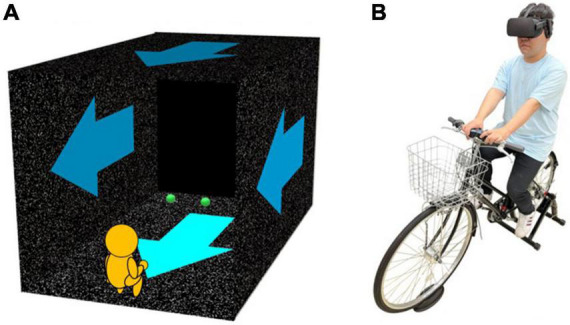
A pattern diagram of the virtual reality environment presented using a head-mounted display **(A)** and the bike **(B)**. **(A)** In the virtual environment, the body was not presented. Participants observed stimuli at the center of a rectangular parallel-piped tunnel. The tunnel was painted with white random dots (density: 15%) on a black background. In the self-motion conditions with optical flow, the white dots moved toward the participants (i.e., blue arrow) and induced forward self-motion perception. In the self-motion conditions without optical flow, the dots remained stationary. In each trial, the fixation point was first presented at 3 m ahead of the participants along the midsagittal plane. When participants’ pressing a start button, the fixation point disappeared. One second later, a pair of green spheres (visual probes, the height of observer’s subjective shoulder) were presented as an aperture for 100 ms 3 m ahead of them. Their task was to judge whether they can pass through the aperture. **(B)** The bike was fixed using the fixed roller. Participants sat on the bike wearing the HMD. They pedaled it at a constant speed (18 or 72 rotations per minute) along with the guide sound, in the self-motion condition with pedaling. In the conditions without pedaling, they remained seated on the bike. The magnetic sensors were placed at the rear wheel of the bike and the rotation number of the rear wheel was recorded for manipulation check.

All visual stimuli were presented on a head-mounted display (HMD; Oculus Rift) from a first-person perspective. A rectangular parallel-piped tunnel comprising a floor, a ceiling, and left and right walls [11.8 m (horizontal) × 10 m (height) × 21 m (depth)] was simulated. The walls were painted with white random dots on a black background (dot diameter: 4.4 cm; wall coverage: 15%). The simulated viewpoint was at the participant’s eye height, which was 5.9 m from both sidewalls and 1.0 m from of the entrance of the tunnel (i.e., 1.0 m inside the tunnel; Figure 1A). Thus, the participants perceived themselves to be located at the center of the tunnel, and to be moving forward when all the dots moved backward. The body and the bike were not presented in the virtual environment. A fixation point (a red sphere; diameter: 3.0 cm) was presented at the height of the participant’s perceived shoulders, which was measured beforehand with a method of adjustment, and 3.0 m ahead along the midsagittal plane. A pair of visual probes (green spheres; diameter: 5.0 cm) representing the aperture width was presented at the same height and distance as the fixation point.

A bike (Figure 1B; wheel size: 27 inches, handle type: all-arounder, handle width: 55 cm, silver leaf) was fixed on the floor using a fixed roller (Indoor Bicycle Training Cycle Trainer, WEIMALL). The cadence was recorded at 70 Hz by magnetic sensors (Grove–Magnetic Switch, Seed Technology) connected with a microcomputer board (Arduino Uno R3).^1^ White noise (70 dBA) was presented to mask the working noise through a loudspeaker (GX-R3X, ONKYO) placed 2 m ahead of the bike. A guide sound (400 Hz pure tone for 50 ms each) was also presented through the same loudspeaker to have participants keep pedaling at a constant pace. A gamepad (Logicool RumblePad 2) was attached at the center of the handlebars as a response device. All stimuli were controlled by a game engine (Unity Technologies, United States) on a computer (NEXTGEAR-NOTE i5730SA1, MouseComputer Co., Ltd., Japan) running on 64-bit Windows 10.

#### Procedure

The experimental design comprised one independent variable: self-motion speed (slow/fast). We had participants pedal the bike at a constant speed of 0.6 Hz (18 rpm) and 2.4 Hz (72 rpm) in the slow and fast conditions, respectively. Correspondingly, the dots on the walls moved backward at a constant speed of 0.75 and 3.0 m/s in the slow and fast conditions, respectively. These self-motion speeds were used to allow participants to comfortably and stably experience self-motion perception in our VR setting. Participants pedaled the bike in all the self-motion conditions. These two types of self-motion conditions were conducted in different sessions and the order was counterbalanced among the participants. The aperture width was individually defined based on the participant’s subjective shoulder width—either –8, 0, 8, 16, 24, or 32 cm margin on both sides relative to the subjective shoulder width. Positive and negative margin values represent larger and smaller aperture widths than the subjective shoulder width, respectively. Each aperture width was presented 10 times in random order in each self-motion condition.

The sessions started with a 20-s pedaling practice. The participants pedaled the bike along with the guide sound and continued pedaling during the session. The background visual stimuli were presented from the beginning of the session. Each trial started with the presentation of the fixation point. The aperture was presented for 100 ms, a second after the participants pressed a start button and the fixation point disappeared. The participant’s task was to judge whether the aperture was passable without bumping and press an appropriate button of the response device. The next trial then began 1 s after the response. The white noise and guide sound were continually presented during each session. Short breaks were introduced between the experimental sessions. About a half-hour was required to complete all the experimental sessions.

#### Statistical analysis

Statistical analyses were performed using JASP (version: 0.13.1.0)^2^ and R (version: 3.6.1). The proportion of “passable” responses was calculated for each margin in each condition. The cumulative gaussian distribution functions were fitted to the individual data. The margin corresponding to a 50% point on the function was defined as the critical margin with which the participant’s perception was changed from passable to impassable.

The cadences for five participants were not properly measured because of malfunction. Thus, we removed these participants from the following cadence-related analyses. To investigate whether the participants accurately pedaled the bike, we first performed a one-sample *t*-test against 18 and 72 rpm in slow and fast self-motion conditions with Holm’s correction, respectively. We also performed a paired *t*-test on the cadence data to investigate whether the pedaling performance differed between the self-motion speed conditions.

Regarding the critical margin data, the Shapiro-Wilk tests revealed the normality of the data. We performed a paired *t*-test to investigate the effect of the self-motion condition. Furthermore, we analyzed the correlation between the critical margin and cadence using Spearman’s correlation analysis. In each analysis, effect sizes were determined using Cohen’s *d* (absolute value).

## Results and discussion

Cadence analyses were performed using the data of 19 participants [mean age: 21.7 ± 0.8 (*SD*) years], with the exception of 5 participants whose cadence were not properly measured due to malfunction. The mean cadences (rpm; ± standard deviation: *SD*) were 22.11 (± 4.80) and 67.78 (± 1.78) in the slow and fast conditions, respectively, which were slightly faster and slower than the requested cadences (18 rpm and 72 rpm) [slow: *t*(18) = 3.74, *p* = 0.002, *d* = 0.86; fast: *t* (18) = 10.34, *p* < 0.001, *d* = 2.37]. However, a significant difference was observed between these conditions [*t*(18) = 37.50, *p* < 0.001, *d* = 8.60]. These cadence analyses suggest our manipulation of self-motion speeds succeeded.

**FIGURE 2:**
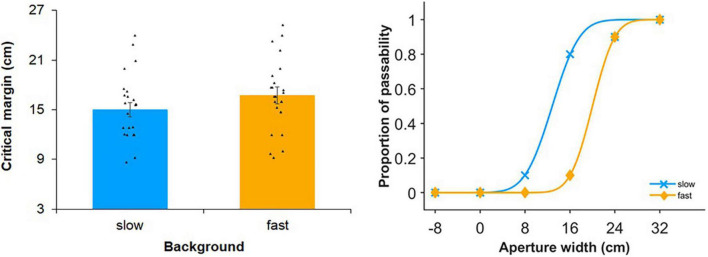
Results of experiment 1. In the left panel, each bar graph represents an average of the critical margin across participants in each self-motion condition. The critical margin was 50% threshold when fitting the cumulative gaussian distribution functions using aperture width conditions in each self-motion condition. Dots indicate individual critical margins in each of self-motion conditions. In the right panel, passable rate of a representative participant in each aperture width condition was plotted and fitted with the cumulative gaussian distribution functions in each self-motion condition. Larger positive values indicate larger margin expansion. Error bars show standard errors.

Figure 2 shows the mean critical margin in each self-motion condition across participants. The *t*-test revealed that the critical margin significantly increased with self-motion speed [*t*(23) = 2.44, *p* = 0.023, *d* = 0.50], suggesting that the effect of speed was observed in the bike riding situation, which was consistent with walking (Warren and Whang, 1987). Additionally, Spearman’s correlation analyses showed no significant correlations between the cadence and critical margin in each of self-motion conditions (slow: ρ = −0.39, *p* = 0.095; fast: ρ = −0.09, *p* = 0.719).

## Experiment 2

Experiment 1 revealed that the increase of self-motion speed expanded the critical margin during the bike riding situation similar to the walking situation (Warren and Whang, 1987). Experiment 2 investigated how visual and non-visual self-motion cues modulated the passable width perception, by comparing background (moving/static) and pedaling (with/without).

### Methods

#### Participants

All participants of Experiment 1 took part in Experiment 2 as well.

#### Apparatus and stimuli

The same apparatus and stimuli as Experiment 1 were used except for the following. Participants pedaled the bike at a constant pace of 1.2 Hz (36 rpm). Correspondingly, the dots on the walls moved backward at a constant speed of 1.5 m/s. The tested aperture widths were the same as used in Experiment 1. Thus, we set the speed between those in Experiment 1 so that the perceived passable width was measurable with the same aperture widths used in Experiment 1.

#### Procedure

##### Critical margin measurements

The experimental design was composed of two independent variables: background (moving/static) × pedaling (with/without). The dots on the walls moved backward at a constant speed of 1.5 m/s in the moving background condition, while they kept static in the other condition. Participants pedaled the bike in the with-pedaling condition, while they kept their feet stationary on the pedals in the without-pedaling condition. Different self-motion conditions were conducted in different sessions (i.e., each self-motion condition was blocked), and the order of the sessions was counterbalanced among the participants. The aperture width was individually defined based on the participant’s subjective shoulder width—either –8, 0, 8, 16, 24, or 32 cm margin on both sides relative to the subjective shoulder width. Positive and negative margin values represent larger and smaller aperture widths than the subjective shoulder width, respectively. Each aperture width was presented 10 times in random order in each self-motion condition.

The with-pedaling sessions started with a 20-s pedaling practice. The participants pedaled the bike along with the guide sound and kept pedaling during the session. In the without-pedaling sessions, the participants sat on the bike and waited for the first 20 s, and subsequently, the first trial began. The background visual stimuli were presented from the beginning of the session. Each trial started with the presentation of the fixation point. The aperture was presented for 100 ms, a second after the participants pressed a start button and the fixation point disappeared. The participant’s task was to judge whether the aperture was passable without bumping and press an appropriate button of the response device. The next trial began 1 s after the response. The white noise and guide sound were continually presented during each session. Short breaks were introduced between the experimental sessions.

##### Depth perception measurements

The perceived depth of the aperture was additionally measured using the same visual stimuli, as in the main experiment, and a method of adjustment. This was to investigate whether changes in the perception of passable width during self-motion simulation were caused by changes in the apparent depth of the aperture by the background motion, and not by the self-motion mechanisms (cf. Watanabe et al., 2004). There were two background sessions: moving and static background sessions. In either session, each trial started with the presentation of the background and fixation point. Five seconds later, the fixation point disappeared. One second after that, the aperture was presented for 100 ms with the participant’s subjective shoulder width. One second later, the background was replaced with random-dot patterns, which was updated at 10 Hz and presented for 1 s to mask the (motion) aftereffects. Immediately after the random-dot patterns disappeared, a visual probe was presented at a random depth position between 2 and 4 m with the static background. The participants’ task was to adjust the probe to the aperture depth by pressing the keys of the response device. The lateral position of the visual probe was matched with either position of the aperture probe (although its initial depth position was varied). The next trial began 1 s after the response. Each participant performed 20 trials in each background condition. This experiment was conducted after the aforementioned main experiment. It took about 1 h to complete all the experimental sessions.

#### Statistical analysis

The cadences for five participants were not properly measured because of malfunction. Thus, we removed these participants from the following cadence-related analyses. With the remaining participants, we investigated whether the participants accurately pedaled the bike and whether the pedaling performance differed between the moving and static background conditions. Thus, in the cadence data, we first performed a one-sample *t*-test against 36 rpm in the with-pedaling conditions with Holm’s correction, and then performed a paired *t*-test between the with-pedaling conditions.

Regarding the critical margin, the Shapiro-Wilk tests revealed the normality of the data. Thus, we performed a two-way repeated measures ANOVA (background × pedaling) on the critical margin data. Furthermore, we investigated the relationship between the critical margin and cadence using Spearman’s correlation analysis.

Regarding the depth perception data, the mean perceived probe depth was calculated in each background condition in each participant, and the difference between the representative value and the actual aperture depth (3.0 m ahead of them) was calculated. The Shapiro-Wilk tests revealed the normality of the data. Thus, we performed a paired *t*-test. In each analysis, effect sizes were determined using Cohen’s *d* (absolute value) or partial Eta squared.

## Results and discussion

Cadence analyses were conducted 19 participants [mean age: 21.7 ± 0.8 (*SD*) years], with the exception of 5 participants whose cadence data were not properly measured due to malfunction. The mean cadences of the pedaling trials were 37.21 (± 2.11) and 37.35 (± 1.88) for the moving and static background conditions, respectively. One-sample *t*-tests against 36 rpm revealed that the cadences were slightly faster than 36 rpm in both conditions [*ts* (18) > 2.50, *ps* < 0.022, *ds* > 0.57]. However, no significant difference was observed between the moving and static background conditions [*t*(18) = 0.21, *p* = 0.835, *d* = 0.05]. These cadence analyses suggest that the participants equally pedaled the bike in both conditions.

**FIGURE 3:**
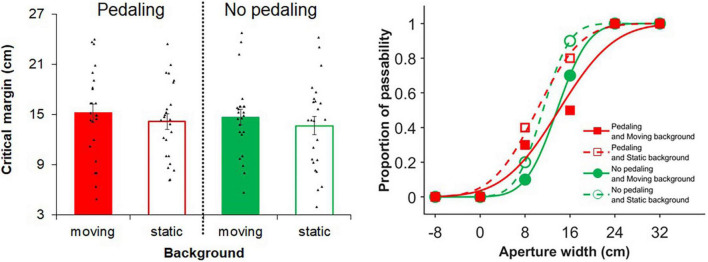
Results of experiment 2. In the left panel, each bar graph represents an average of the critical margin across participants in each self-motion condition. The critical margin was 50% threshold when fitting the cumulative gaussian distribution functions using aperture width conditions in each self-motion condition. Dots indicate individual critical margins in each of self-motion conditions. In the right panel, passable rate of a representative participant in each aperture width condition was plotted and fitted with the cumulative gaussian distribution functions in each self-motion condition. Larger positive values indicate larger margin expansion. Error bars show standard errors.

Figure 3 shows the mean critical margin in each self-motion condition across participants. The two-way ANOVA on the critical margin data revealed a significant main effect of background [*F*(1, 23) = 6.76, *p* = 0.016, η*_*p*_*^2^ = 0.23], suggesting that visual self-motion information can expand the critical margin. The ANOVA also showed no significant main effect of pedaling and the background × pedaling interaction [pedaling: *F*(1, 23) = 0.87, *p* = 0.361, η*_*p*_*^2^ = 0.04; background × pedaling interaction: *F*(1, 23) < 0.01, *p* = 0.980, η*_*p*_*^2^ < 0.01]. Additionally, Spearman’s correlation analyses showed no significant correlations between the cadence and critical margin (moving with pedaling: ρ = −0.01, *p* = 0.968; static with pedaling: ρ = −0.31, *p* = 0.193).

**FIGURE 4:**
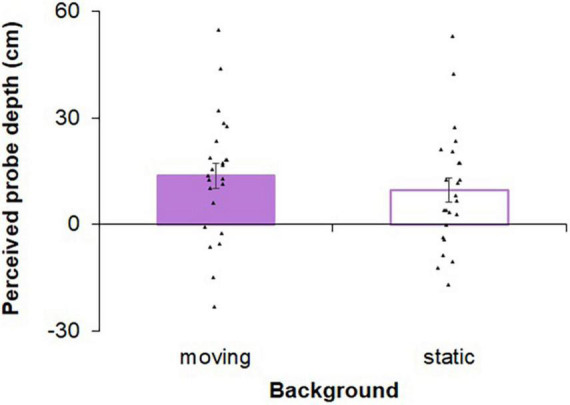
Results of the perceived depth of the aperture in the additional experiment. Each bar graph represents an average of the differences from the simulated probes’ depth (i.e., 3.0 m ahead of them) across participants in each background condition. Dots indicate individual critical margins in each of the self-motion conditions. Larger positive and negative values indicate farther and nearer probes perception from the simulated probes’ depth, respectively. Error bars show standard errors.

Figure 4 shows the mean depth perception data in each background condition across participants in the additional experiment. Paired *t*-test revealed no significant difference between these conditions [*t* (23) = 1.66, *p* = 0.111, *d* = 0.34], suggesting that the optical flow had little effects on the perceived depth of the probe in this study.

Warren and Whang (1987) suggests that the perceived passable width is larger during walking than standing still. Our study extends this finding to a different form of locomotion, which is bike riding. The results showed that enlargement of the perceived passable width seemed to be found when the visual information was presented. This suggests that the visual cue during self-motion could contribute to this phenomenon, supporting the dominance of visual cue compared to no-visual cue during bike riding (Sun et al., 2004).

## General discussion

This study investigated the passable width perception in a virtual bike riding situation. Experiment 1 showed that the perceived passable width during bike riding expanded with self-motion speed. Experiment 2 demonstrated that visual self-motion cues mainly contributed to this expansion of perceived passable width. These results suggest that online self-motion information could be utilized to perceive passable width in vehicle locomotion using a VR bike riding situation.

The present study extends the previous finding in a walking situation (Warren and Whang, 1987) to a bike riding situation, which provided direct evidence that online self-motion information contributed to the perception of action possibility constrained by the surrounding environment. Specifically, although Warren and Whang (1987) argued that the difference in estimated passable width between walking (Experiment 1) and standing still (Experiment 2) was due to the way of instruction, our results suggest that this discrepancy could be caused by the existence of online self-motion information.

Studies have shown that visual information plays a dominant role in the perception of the direction and speed of self-motion (e.g., Brandt et al., 1973), and in particular, some perception during bike riding can be affected by visual rather than non-visual cues (Sun et al., 2004). Self-motion speed and direction are provided by visual information in a bike riding situation, wherein bikes often have a gear and the speed is not determined by pedaling the bike, and the direction does not depend on pedaling it. Additionally, Experiment 2 showed that visual self-motion information expanded the critical margin. Thus, visual information is likely to play an important role in the construction of affordance perception during bike riding. We speculate that visual self-motion signal alone is enough to trigger an internal self-motion model which has been acquired by past experience in a biking situation. This model would consequently define the safety margin in order to avoid contacts or collision to the surrounding environment in a given self-motion situation. This explanation is consistent with previous findings that the perceived minimum passable width was enlarged for children (Wilmut and Barnett, 2011), older adults (Hackney and Cinelli, 2011), and patients with Parkinson’s disease (Cohen et al., 2011).

In the studies by Higuchi and his colleagues (Higuchi et al., 2004, 2009), novice wheelchair users underestimated the critical aperture width to pass through for wheelchair use even after the 8-day training for passing through apertures using a wheelchair (Higuchi et al., 2004). Contrarily, expert wheelchair users accurately estimated the spatial requirements for wheelchair use, even while using an unfamiliar wheelchair (i.e., a wheelchair which was not owned by the individual) (Higuchi et al., 2009). These results suggest that the participant’s familiarity to the given locomotion type can affect the perception of passable width as well as the adaptability to a new situation. Based on the post-interviews in the present study, all participants were familiar with bike riding to some extent. Some participants used their bike almost every day (i.e., for commuting purpose), whereas others sometimes used it or had previously used it but currently did not. Such accumulated experiences in bike riding would likely lead to a relatively accurate passable width perception, even though the bike used in the experiments was not the individual’s own. Familiarity to bike riding in this study may be related to the fact that we used a popular bike. In order to clarify mechanisms underlying individual differences in the perceived passable width and adaptability to a new tool, future research should quantify the individual’s familiarity to a given locomotion type and tool.

Relevant to safety margin, peripersonal space is suggested to be related to defensive behavior to avoid contact with objects in the environment (Graziano and Cooke, 2006). Peripersonal space (PPS) is the space immediately around the body (Rizzolatti et al., 1981a,b) that contributes to interactions with other people and objects in the environment. Most behavioral studies have defined the PPS boundary as the farthest distance at which visuo-tactile or audio-tactile interaction occurs. Using this definition, studies have reported that the range of PPS is changeable depending on online self-motion information. Noel et al. (2015) suggested PPS expansion during walking on treadmill. Other studies have suggested that PPS modulation during self-motion can occur with either proprioceptive information alone (Amemiya et al., 2019) or visual information alone (Kuroda and Teramoto, 2021). Recently, Kuroda and Teramoto (2022) suggested that PPS expanded more in pedaling a bike with optical flow than optical flow alone during virtual bike riding. This indicates the importance of both visual and non-visual information in PPS modulation during bike riding. Despite the commonality in phenomenal aspects and assumed roles between the passable width perception and PPS (both assume to be related to defensive behavior), current results showed that the perceived passable width expanded only when visual self-motion cue was presented. The phenomenal discrepancy between PPS and passable width perception was also reported in wheel chair use. While novice wheelchair users exhibited underestimation of the critical aperture width (Higuchi et al., 2004), PPS expanded at 13 min of passive wheelchair experience, wherein an experimenter moved the wheelchair the participant sat on (but not after active use) (Galli et al., 2015). Including current results, these studies suggest that the passable width perception could be based on an independent mechanism of PPS. In order to clarify this matter, however, future studies need to investigate passable width perception and PPS (visuo-tactile or audio-tactile interaction) using the same experimental setups and participants.

Despite these findings, the present study had three limitations. First, the data were obtained in the virtual environment. We adopted a virtual bike riding situation for controllability and safety. However, the distortion of virtual space has often been reported (e.g., Loomis et al., 1992). Absolute distances or widths in this study must be interpreted with caution, while the differences between conditions were reliable. Second, it is not clear how handlebar width affected the current performance on passable width perception. Although the handlebar width was larger than the participants’ shoulder widths, their hands were always on the response device attached at the center of the handlebar and the participants could not directly see their hand during the task due to the HMD. Therefore, we believe that passable width perception was based on the shoulder width. However, grabbing the tips of the handlebar might modulate the passable width perception in a bike riding situation. Lastly, horizontal sway with pedaling was not measured. In a natural bike riding situation, the horizontal sway of the bike would likely affect the passable width perception, wherein a larger sway would induce a larger critical margin. However, in the current experimental setup, the bike was fixed on the floor, which prevents the actual sway of the bike. Furthermore, the horizontal sway of the participants’ shoulders hardly occurred due to relatively low pedal loading. In such a situation, the pedaling effect was not significant, suggesting that non-visual self-motion information may not be so important for passable width perception during bike riding. Future studies should investigate the effect of the horizontal sway on the passable width perception during bike pedaling.

## Conclusion

In conclusion, this study investigated the effects of online self-motion information on passable width perception during bike riding. The results showed that the perceived passable width increased with increasing self-motion speed and when visual self-motion information was presented. These results suggest that online self-motion information, especially derived from the visual system, contributes to the passable width perception during bike riding.

## Data availability statement

The raw data supporting the conclusions of this article will be made available by the authors, without undue reservation.

## Ethics statement

The studies involving human participants were reviewed and approved by the Ethics Committee of the Graduate School of Humanities and Social Sciences, Kumamoto University. The patients/participants provided their written informed consent to participate in this study.

## Author contributions

NK, KI, and WT developed the study concept, contributed to the study design, data collection, analysis, and data interpretation, and drafted the manuscript. All authors approved the final version of the manuscript and agreed to be accountable for all aspects of the work in ensuring that questions related to the accuracy or integrity of any part of the work are appropriately investigated and resolved.

## Conflict of interest

The authors declare that the research was conducted in the absence of any commercial or financial relationships that could be construed as a potential conflict of interest.

## Publisher’s note

All claims expressed in this article are solely those of the authors and do not necessarily represent those of their affiliated organizations, or those of the publisher, the editors and the reviewers. Any product that may be evaluated in this article, or claim that may be made by its manufacturer, is not guaranteed or endorsed by the publisher.

## Footnotes

1
http://arduino.cc

2
https://jasp-stats.org/
